# Genetic overlap between Alzheimer’s disease and immune‐mediated diseases: An atlas of shared genetic determinants and biological convergence

**DOI:** 10.1002/alz.089871

**Published:** 2025-01-03

**Authors:** Nitesh Enduru, Brisa S. Fernandes, Shahram Bahrami, Yulin Dai, Ole Andreassen, Zhongming Zhao

**Affiliations:** ^1^ School of Biomedical Informatics, The University of Texas Health Science Center at Houston, Houston, TX USA; ^2^ School of Public Health, The University of Texas Health Science Center at Houston, Houston, TX USA; ^3^ Oslo University Hospital & Institute of Clinical Medicine, University of Oslo, Olso, Olso Norway; ^4^ NORMENT Centre, Division of Mental Health and Addiction, Oslo University Hospital, Oslo, Olso Norway

## Abstract

**Background:**

An increasing body of evidence has suggested that the pathogenesis of Alzheimer’s disease (AD) is not confined to the neurons but instead that neuroinflammation plays a significant role in the disease, with an interplay between the brain and the immune system. So far, their shared genetic components have not been systematically studied.

**Method:**

We investigated the shared genetic architecture between AD and a plethora of immune‐mediated diseases using the genome‐wide association studies (GWAS) summary statistics data: allergic rhinitis, asthma, atopic dermatitis, celiac disease, Crohn’s disease, hypothyroidism, primary sclerosing cholangitis, RA, systemic lupus erythematosus, ulcerative colitis, and vitiligo. We developed a pipeline for global and local genetic correlation analysis between diseases and quantifying the shared loci among diseases using MiXeR. We used the conditional/comjunctional FDR to identify the shared loci and, finally, performed bi‐directional MR to assess the causal relationship between AD and the 11 immune‐mediated diseases.

**Result:**

Our results unveiled a significant genetic overlap between AD and 11 individual immune‐mediated diseases despite negligible genetic correlations, suggesting a complex shared genetic architecture distributed across the genome. With conjFDR <0.05, we identified 76 shared genomic loci. After excluding the 6 loci jointly associated with more than one pair of diseases, we had 70 unique shared genomic loci between AD and immune‐mediated diseases. Among them, 38 loci were novel for AD only, 25 were novel for immune‐mediated diseases only, and 17 were novel to both AD and immune‐mediated diseases. The shared loci between AD and immune‐mediated diseases implicated several genes, including *GRAMD1B*, *FUT2*, *ADAMTS4, HBEGF, WNT3, TSPAN14, DHODH, ABCB9* and *TNIP1*, all of which are protein‐coding genes and thus potential drug targets. Enrichment analysis of these shared genes revealed pathways related to the immune system and cell adhesion. Finally, *in silico* single‐cell analyses showed enrichment of immune and brain cells, including neurons and microglia.

**Conclusion:**

Our results suggest a genetic relationship between AD and the 11 immune‐mediated diseases, pinpointing the existence of a shared however non‐causal genetic basis. These identified protein‐coding genes have the potential to serve as a novel path to therapeutic interventions for both AD and immune‐mediated diseases and their comorbidities.

